# Effects of Wooden Breast Syndrome in Broiler Chicken on Sarcoplasmic, Myofibrillar, and Connective Tissue Proteins and Their Association with Muscle Fiber Area

**DOI:** 10.3390/foods12183360

**Published:** 2023-09-07

**Authors:** Binbin Li, Jere Lindén, Eero Puolanne, Per Ertbjerg

**Affiliations:** 1 Department of Food and Nutrition, University of Helsinki, 00014 Helsinki, Finland; binbin.li@helsinki.fi (B.L.); eero.puolanne@helsinki.fi (E.P.); 2 Department of Veterinary Biosciences, University of Helsinki, 00014 Helsinki, Finland; jere.linden@helsinki.fi; 3 Finnish Centre for Laboratory Animal Pathology (FCLAP), Helsinki Institute of Life Science (HiLIFE), University of Helsinki, 00014 Helsinki, Finland

**Keywords:** woody breast, muscle protein fraction, myofiber area, myodegeneration, protein denaturation

## Abstract

This study was conducted on chicken *pectoralis major* muscle with different wooden breast severity in combination with different sampling locations to investigate the effects of wooden breast syndrome on protein traits and total myofiber area, and their associations. Contents of sarcoplasmic, salt-soluble myofibrillar and salt-insoluble protein and proportion of total myofiber area significantly declined with increasing severity in the superficial part of muscle, whereas the amount of heat-soluble/insoluble collagen and protein denaturation as well as the area of degenerated myofibers, connective tissue and cellular infiltrates increased. Myofibril protein content indicators showed strong positive correlations to total myofiber area. Moreover, PCA results indicated that severe wooden breast is positively linked to muscle collagen content and to protein denaturation. Our results suggest that decrease in sarcoplasmic and myofibrillar proteins is associated with reduction of myofiber area. In turn, the muscle fibers are replaced by connective tissue, accompanied by excessive myofibrillar and sarcoplasmic protein denaturation.

## 1. Introduction

During the last decade, the prevalence of the abnormality described as wooden (or woody) breast and its effects on meat quality have received extensive attention from meat and animal scientists. Although the etiopathogenesis of this myopathy has not yet been elucidated, many studies have demonstrated that the rapid growth rate and high breast yield in chickens are closely associated with the onset and development of muscle abnormalities such as wooden breast [1,2]. In comparison to normal muscles, the wooden-breast-affected muscles exhibit, macroscopically, paleness, with areas that bulge out and have a hardened consistency. Histologically, wooden breast muscles show chronic myodegeneration with regeneration, interstitial edema, as well as the accumulation of loose connective tissue and fibrosis [3]. The wooden breast lesions are generally symmetrical, however, the superficial part of the breast muscle has been found to be more vulnerable to wooden breast lesions than the deep part [4]. In addition to the above-mentioned conspicuous macroscopic and histological alterations, the disease also shows substantial negative effects on meat quality, including a poor water-holding capacity [5], altered textural properties with increased hardness [6], as well as decreased nutritional value with increased lipid and protein oxidation together with greater amounts of fat and collagen and less total protein [7], which would cause a low customer acceptance and, thus, economic loss.

A significant decrease in protein content and an increase in collagen in wooden breast muscle has been widely reported, and these profound changes are likely related to the progressive myodegeneration, deposition of interstitial connective tissue, and fibrosis [3,8,9,10]. Generally, muscle protein can be classified into three groups: sarcoplasmic, myofibrillar, and stromal proteins [11]. A reduction in the total protein to collagen ratio may lead to a series of changes in the functional properties of the breast muscle and the technological properties of the meat. Both Mudalal et al. [12] and Geronimo et al. [13] reported that a significant increase in cooking loss and a lower water-holding capacity may be linked to the decrease in total protein content in affected muscles, especially a reduction in the contents and solubility of sarcoplasmic and myofibrillar proteins, which are considered to play a crucial role in the functional characteristics of meat products. Moreover, higher levels of compression values, shear force, hardness, and chewiness in raw wooden breast muscles may be a consequence of fibrosis, and thus, of the abundance of collagen [4,14]. Mutryn et al. [15] found that a relevant gene for collagen formation was upregulated in wooden breast muscle. However, the hardness is not only associated with the collagen content, but also with the organization of the fibrillar collagen, such as crosslinks which mature and become non-reducible in various degrees [16]. Although some studies involving the changes in the protein and collagen amounts in wooden breast muscles have been conducted, available knowledge on the level and properties of muscle protein fractions in relation to the wooden breast condition is limited. Therefore, this study aims at a better understanding of the underlying mechanisms behind changes in proteins associated with muscle structure alterations in wooden breast muscle.

The superficial layer of wooden breast muscle shows myodegeneration and connective tissue accumulation [4], the sarcomere length is longer [17], and the proteins are more prone to oxidation [7] compared to those in the deep layer. However, specific effects on the muscle proteins are not well elucidated, and information on the relative effects in the superficial versus the deep layer is lacking. This study was conducted to evaluate the effects of different wooden breast severities (normal, mild, moderate, and severe) and sampling locations (superficial and deep part) on the contents of muscle protein fractions (sarcoplasmic, salt-soluble myofibrillar and salt-insoluble protein, and heat-soluble/insoluble collagen). In addition, the proportions of muscle subsection areas (total myofiber area versus interstitial connective and adipose tissue + necrosis area) were evaluated using histological image analysis to study whether the protein compositional changes are directly related to the cross-sectional areas of histological alterations in muscles. Intrinsic fluorescence intensity and surface hydrophobicity, to reflect sarcoplasmic and myofibrillar protein denaturation, respectively, were also measured. In order to obtain new insight on the changes in muscle sub-fractions, correlations of the above parameters were established by applying the Pearson correlation method and principal component analysis.

## 2. Materials and Methods

### 2.1. Sample Processing

After hatching, a total of 171 Ross 308 male broilers were allocated to 8 pens in the Laboratory Animal Centre at the University of Helsinki farm facilities in Viikki. Birds were fed ad libitum on a standard diet, and the temperature and lighting were controlled according to the Ross breeders’ manual. Fifty-six birds were randomly selected at the age of 41 d, and slaughtered in accordance with EC Regulation No 1099/2009. The left *pectoralis major* muscle was excised and palpated immediately after termination. Based on the palpation, in total 40 muscles were selected and allocated to the following 4 categories (*n* = 10 per category) for the wooden breast severity: muscle without wooden breast condition (normal); mildly affected (mild); focally or diffusely affected (moderate); pronounced hardness diffusively throughout the muscle (severe).

Samples were obtained from the middle area of the left breast muscle immediately after the termination, wrapped with aluminum foil, and frozen in liquid nitrogen. Then, the samples were stored at −80 °C until analysis.

All the indicators were investigated in both the superficial and deep part of each fil-let. After removing the surface skin (upper 2 mm) of the breast muscle, the first 10 mm was defined as the superficial part and the deeper 10 mm was defined as the deep part, and the deep part was taken 1–2 mm away from the superficial part.

### 2.2. Muscle Protein Fraction Extraction and Measurement

#### 2.2.1. Extraction of Sarcoplasmic Fraction

The sarcoplasmic protein fraction was obtained according to the method of Liu et al. [18]. In detail, 0.5 g of the frozen breast sample was homogenized with 10 mL of precooled extraction buffer (75 mmol/L KCl, 10 mmol/L KH_2_PO_4_, 2 mmol/L MgCl_2_, 2 mmol/L EGTA, pH 7.0) using an IKA Ultra-Turrax T25 homogenizer (Labortechnik, Staufen, Germany) at 13,500 rpm for 20 s. Then, the homogenate was centrifuged at 10,000× *g* at 4 °C for 10 min and the supernatant collected. Another 10 mL of precooled extraction buffer was added to the pellet and after homogenizing with the same homogenizer at the same speed for 10 s and centrifuging, the supernatant was collected and mixed with the previous one, which was considered as the sarcoplasmic fraction. Then, the protein content was determined using an RC DC protein assay kit (Bio-Rad Laboratories, Hercules, CA, USA) using BSA as the standard.

#### 2.2.2. Isolation of Salt-Soluble Myofibrillar and Salt-Insoluble Protein Fractions

The pellet, which mainly contains myofibrils, was washed with 10 mL extraction buffer by homogenizing at 13,500 rpm for 10 s and centrifuging at 10,000× *g* at 4 °C for 10 min. After two times of washing, the precipitation part was re-suspended in 5 mL of cold sodium phosphate buffer (50 mmol/L, pH 7.5) with 0.6 mmol/L NaCl, and homogenized at 13,500 rpm for 30 s. Then, the homogenate was kept at 2 °C for 2 h followed by centrifuging at 12,800× *g*, at 4 °C for 10 min. The pellet was washed once again by using 5 mL of the same cold buffer. The supernatants from these two-time extractions were combined and defined as the salt-soluble myofibrillar fraction. After rinsing twice with sodium phosphate buffer (50 mmol/L, pH 7.5), the precipitate was regarded as the salt-insoluble protein fraction. The protein contents of the salt-soluble and salt-insoluble fractions were measured using the protein assay kit as above.

#### 2.2.3. Determination of Heat-Soluble and Heat-Insoluble Collagen

The contents of heat-soluble and heat-insoluble collagen were determined by determining the hydroxyproline concentration of hydrolysates based on the method of Fang et al. [19], Goll et al. [20], and Woessner Jr [21] modified by Voutila et al. [22]. One gram of minced meat in duplicate, was homogenized with 4 mL Ringer’s solution containing 147 mmol/L NaCl, 4 mmol/L KCl, and 2.2 mmol/L CaCl_2_ in a 50 mL centrifuge tube and was then heat treated in a 77 °C water bath for 65 min with gentle shaking every 15 min. After the mixture was centrifuged at 4300× *g* for 10 min, the supernatant was poured into a digestion tube. A volume of 2 mL of Ringer’s solution was added to the precipitate to rinse it, then it was centrifuged again and the supernatant was combined with that of the previous step. The final pellet was transferred into another digestion tube. After that, digestion tubes with supernatants and pellets were hydrolysed with 9 mL of 3 mol/L sulphuric acid at 110 °C for 16 h in a digestion block (LabtecTM DT220 Digestor, FOSS Analytical Co., Hillerød, Denmark). Then, the hot hydrolysates were diluted to 20 mL with distilled water. Ten mL of the filtered solution (5 mL for insoluble collagen) was neutralised to pH 6.0 and brought to a final volume of 25 mL by using 3 mol/L NaOH and distilled water, respectively. L-hydroxyproline was used to establish a standard curve with a range from 0.5 to 4 μg hydroxyproline/mL. The hydroxyproline content in the samples was measured using a colorimetric reaction. Firstly, 5 mL of the sample solution was mixed with 2.5 mL chloramine-T buffer (pH 6.0) which contained citric acid monohydrate, NaOH, CH_3_COONa, 1-propanol, and kept at room temperature for 30 min to oxidize the hydroxyproline. Then, the color of the oxidized solution was developed after the addition of 2.5 mL DMABA (4-dimethylaminobenzaldehyde) and incubation for 30 min at 60 °C followed by cooling to room temperature. The color intensity was read spectrophotometrically at 560 nm. The hydroxyproline content in the supernatant and precipitate was converted to the content of heat-soluble and heat-insoluble collagen, respectively, in chicken *pectoralis major* muscle by using a conversion factor of 7.25.

### 2.3. Histological Analysis

Histological analysis was performed on each wooden breast category for a total of 38 breasts: the numbers of muscles in the normal, mild, moderate, and severe categories were 9, 10, 10, and 9, respectively. Transversal histological samples, measuring approximately 2.5 cm (ventral surface–deep dorsal muscle) × 2 cm (medial–lateral), were obtained from the thickest part (immediately cranially from the protein sample) of the left breast muscle. The samples were fixed in 10% neutral buffered formalin, embedded in paraffin, sectioned at 4 µm thickness, and stained with haematoxylin and eosin (HE).

**Figure 1:**
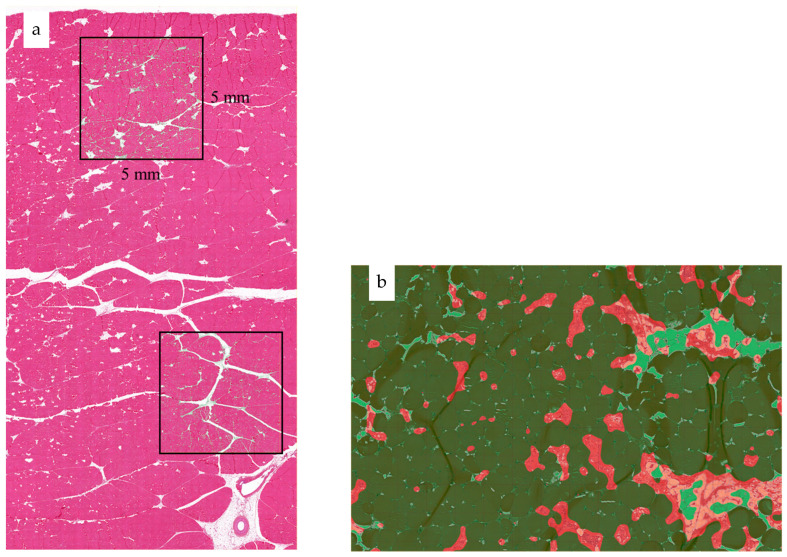
Selection of representative region of interest (square, 5 mm × 5 mm) in the superficial and deep part of each sample muscle (**a**) and the image for total myofiber area and connective tissue + necrosis area calculation (**b**) by employing QuPath version 0.30.

The HE-stained slides were scanned to whole slide images (WSIs) using a 3D HISTECH Pannoramic 250 Flash III scanner (3DHISTEC, Budapest, Hungary) with 20× objective, and the WSIs were analyzed applying the QuPath software, version 0.30 [23]. In general, two 5 mm × 5 mm (25,000,000 µm^2^) region of interest (ROI) squares were manually drawn on the superficial and deep parts on each WSI as the surface ROI and deep ROI (Figure 1a). The uppermost side of the surface ROI was approximately 1 mm away from the ventral surface of the breast muscle, and the uppermost side of the deep ROI was more than 10 mm away from the muscle ventral surface.

Inside the ROIs, white translucent areas, representing adipose tissue or interstitial edema, were first excluded, employing a thresholder (set to 192 with smoothing sigma parameter 2.5) that segmented the ROIs into “ignored” and “tissue” classes. In the second phase, “muscle fiber” areas and “connective tissue + necrosis” areas, representing loose connective tissue, fibrosis, and degenerated or regenerating muscle fiber cross-sections, were segmented and measured based on hematoxylin stain intensity (threshold 0.04, sigma 10.0), excluding the “ignored” class areas. Both threshold and sigma values were initially set manually based on how well they visually separated the tissue components in the first three WSIs (six ROIs), and after that all ROIs were run by using the same values. The analyses were performed blinded to the macroscopic classification of the wooden breast severity.

The percentages of total myofiber area (the area occupied by undamaged myofibers, shown in Figure 1b as dark green) per ROI and connective tissue + necrosis area per ROI were calculated as:

$$Total\;myofiber\;area\left(\%\right)=\frac{muscle\;fiber\;area\;in\;the\;tissue\;class\;\left({\mu m}^{2}\right)}{25,000,000\;({\mu m}^{2})}\times 100\%$$

$$Connective\;tissue\;+\;necrosis\;area\;\left(\%\right)=\frac{connective\;tissue+necrosis\;area\;in\;the\;tissue\;class\;({\mu m}^{2})}{25,000,000\;({\mu m}^{2})}\times 100\%$$

### 2.4. Protein Denaturation Measurements

Protein denaturation measurements on sarcoplasmic and myofibrillar fractions of each breast were carried out by measuring intrinsic fluorescence emission spectra and surface hydrophobicity as indexes, respectively, as previously described by Zhang and Ertbjerg [24]. The intrinsic fluorescence spectrum of tryptophan was measured by applying an LS 55 luminescence spectrometer (PerkinElmer Inc., Waltham, MA, USA) with a final protein concentration of 0.015 mg/mL. The BPB (bromophenol blue) method was here adopted to investigate the surface hydrophobicity of the myofibrils. Briefly, myofibrils were isolated and 1 mL of suspension (adjusted to 2 mg protein per mL) was mixed with 80 μL of 1 mg/mL BPB. After incubation for 10 min at room temperature and centrifugation at 10,000× *g* for 3 min, the absorbance at 595 nm of the 10 times diluted supernatant was recorded, and the results were expressed as μg bound BPB/mg protein.

### 2.5. Statistical Analysis

Statistical analysis was carried out using the IBM SPSS Statistics 26 program. Protein fraction contents and denaturation indicators were analyzed for the 10 birds of each category. For each parameter, duplicates were run for each sample at each location (superficial and deep part). Triplicates were performed on each protein measurement. The effects of wooden breast severity and muscle location (superficial or deep) as well as their interaction on the measured parameters was evaluated by two-way ANOVA using the general linear model with wooden breast severity and location as fixed factors. Parameters showing a significant (*p* < 0.05) effect of the wooden breast severity were further assessed with Duncan’s multiple range test. Pearson correlation coefficients and PCA (principal component analysis) were calculated and analyzed to find the potential associations between parameters.

## 3. Results

### 3.1. Protein Fractions

**Figure 2:**
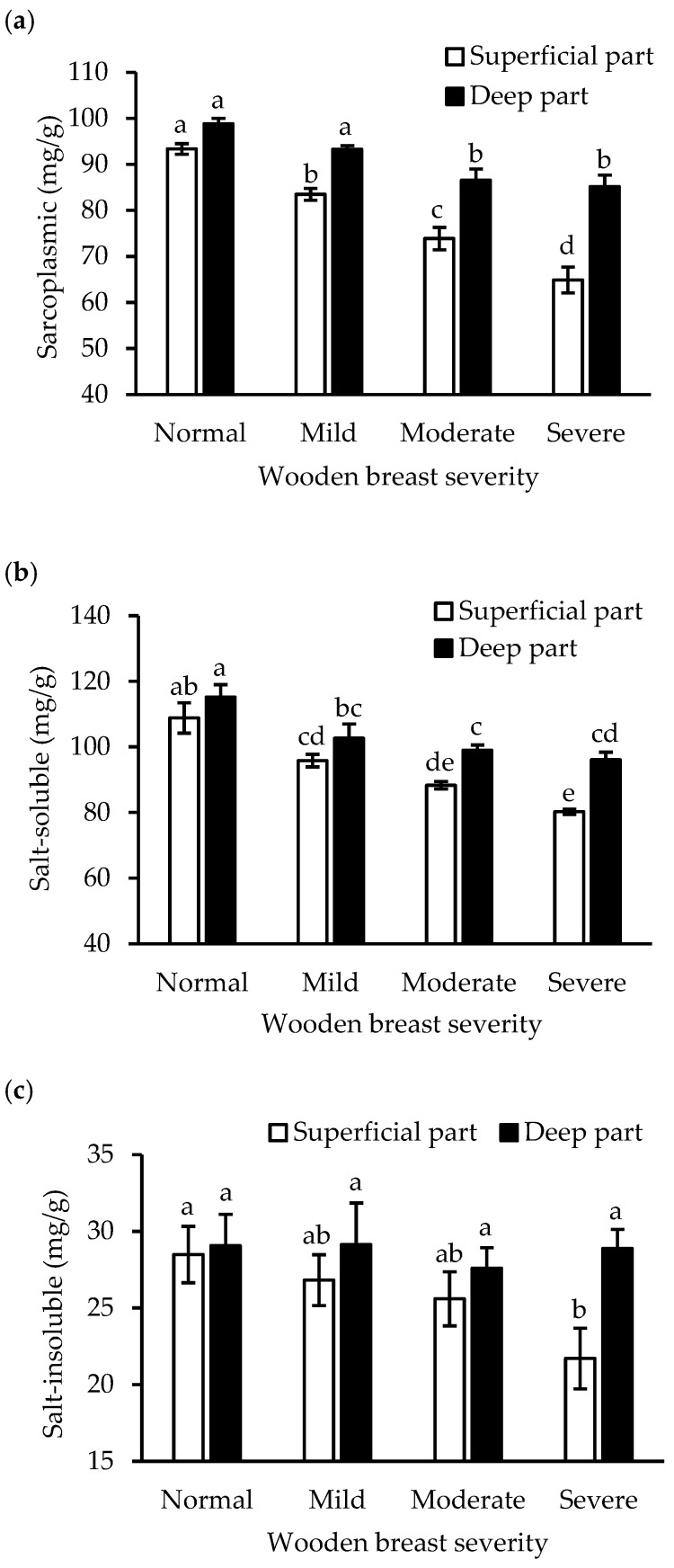
Effects of wooden breast severity on the content of sarcoplasmic protein (**a**), salt-soluble myofibrillar protein (**b**), and salt-insoluble protein (**c**) in the superficial and deep part of *pectoralis major* muscle. Means ± standard errors are shown (*n* = 10). a–e: within each fraction, mean values with the same letter do not differ (*p* > 0.05).

Although data are emerging that wooden breast condition leads to less protein in the *pectoralis major* muscle, it has not been elucidated how sarcoplasmic, myofibrillar, and connective tissue proteins are affected. Both wooden breast severity and location (surface vs. deep part) showed significant main effects on the contents of protein fractions, and all the protein fractions in the superficial part were strongly reduced by wooden breast syndrome (*p* < 0.05, Table 1 and Figure 2). With increasing wooden breast severity, the mean contents of sarcoplasmic protein in the superficial part showed a 30% decrease (*p* < 0.01), from 93 to 65 mg/g muscle, while the corresponding change in the deep part was less pronounced, from 99 to 85 mg/g muscle (Figure 2a). In agreement, a significant interaction effect between severity and location was observed (*p* < 0.01, Table 1). Comparably, the wooden breast severity-related decrease in salt-soluble myofibrillar protein was more pronounced in the superficial part than in the deep part, and the lowest content (80 mg/g muscle) was found in the superficial part of the severely affected muscles (Figure 2b). In addition, both wooden breast severity and location also affected (*p* < 0.05) the content of salt-insoluble protein, which decreased significantly (*p* < 0.05) in the superficial part (from 29 mg/g to 22 mg/g muscle in severe wooden breast) but showed no change in the deep part (Figure 2c).

### 3.2. Heat-Soluble and -Insoluble Collagen

**Figure 3:**
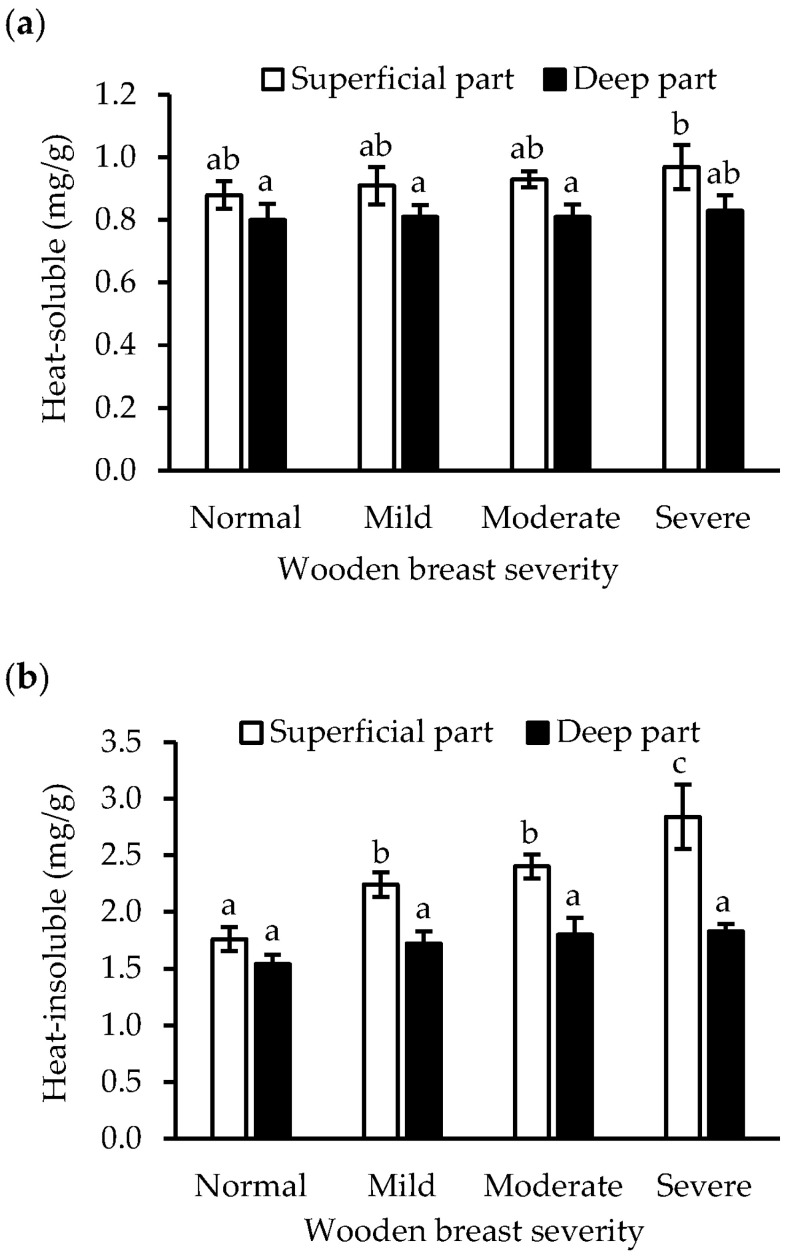
Effects of wooden breast severity on the content of heat-soluble collagen (**a**) and heat-insoluble collagen (**b**) in the superficial and deep part of *pectoralis major* muscle. Means ± standard errors are shown (*n* = 10). a–c: within each fraction, mean values with the same letter do not differ (*p* > 0.05).

Collagen solubility has been linked to the development of mature crosslinks. In this study, wooden breast severity had no effect on heat-soluble collagen (*p* > 0.05), while its concentration was significantly (*p* < 0.01) affected by the sampling location (Table 1). However, in severely affected muscle, the heat-soluble collagen in the superficial part was larger than in the deep part of less affected muscle (*p* < 0.05, Figure 3a). Heat-insoluble collagen contents increased (*p* < 0.01) with wooden breast severity, and the wooden breast severity and muscle location showed significant interaction (*p* < 0.05, Table 1). In normal muscle, the heat-insoluble collagen in the superficial and deep parts did not differ (*p* > 0.05). On the contrary, the heat-insoluble collagen content increased in the superficial parts as wooden breast severity increased: normal < mild = moderate < severe (*p* < 0.05, Figure 3b).

### 3.3. Histological Analysis

**Figure 4:**
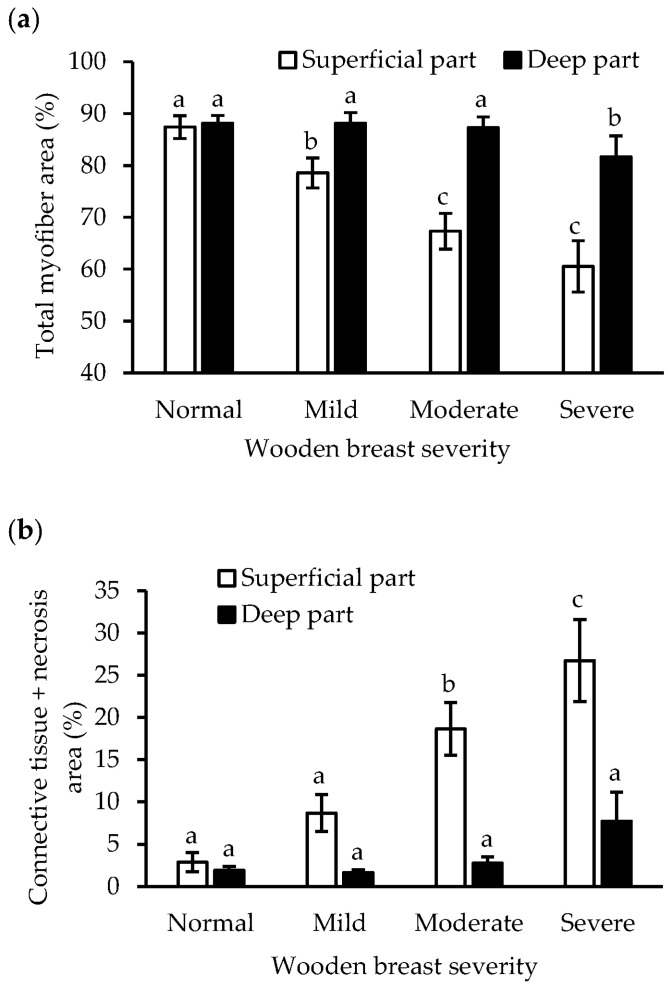
The proportion of total myofiber area (**a**) and connective tissue + necrosis area (**b**) in the superficial and deep part of *pectoralis major* muscle with different wooden breast severity. Means ± standard errors are shown. The number of breasts analyzed in normal, mild, moderate, and severe conditions are 9, 10, 10, and 9, respectively. a–c: within each area, mean values with the same letter do not differ (*p* > 0.05).

**Figure 5:**
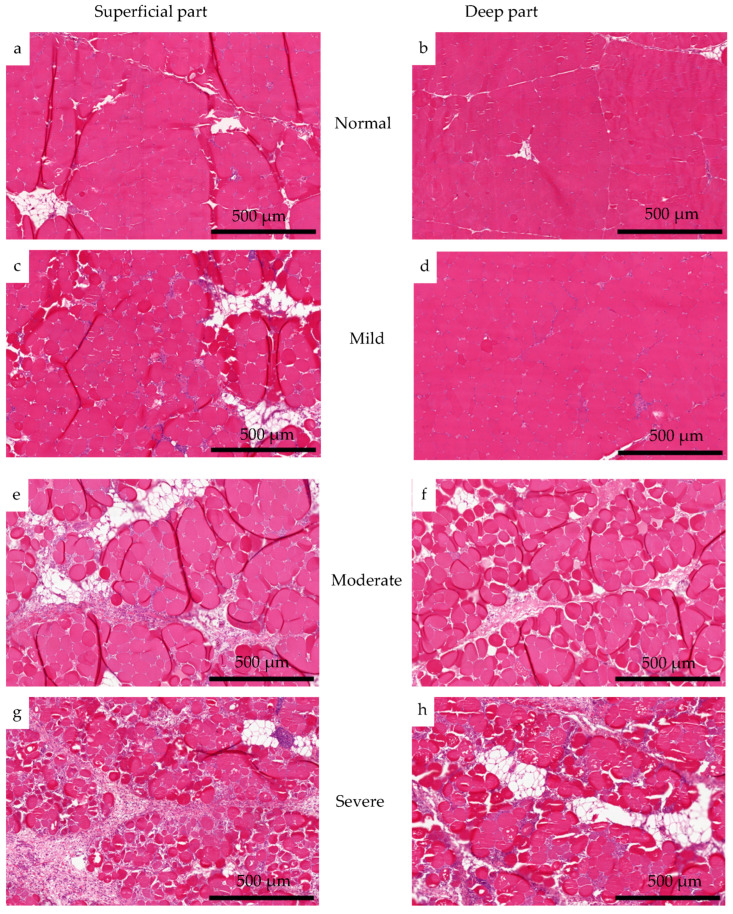
Histological images of superficial and deep part of normal (**a**,**b**) muscles, and muscles affected by wooden breast with mild (**c**,**d**), moderate (**e**,**f**), and severe (**g**,**h**) degree. Stained by hematoxylin and eosin.

It has not previously been analyzed how the decline in muscle fiber proteins and increase in collagen are associated with histological appearance. Total myofiber and connective tissue + necrosis areas were separately measured from the superficial and deep muscle tissue parts of each sample, and both wooden breast severity and sampling location showed significant (*p* < 0.01) main effects and interaction effects on the histological parameters (Table 1). Accordingly, the proportional total myofiber area decreased and the proportional connective tissue + necrosis area increased as wooden breast severity worsened (Figure 4 and Figure 5).

In normal muscles, the proportional total myofiber (87% superficial, 88% deep) and connective tissue + necrosis (2.9% superficial, 1.9% deep) areas showed no difference between the superficial and deep parts (Figure 4). The muscle fibers were tightly packed and generally of equal size, and a small amount of connective tissue and fat was present (Figure 5a,b). In contrast, with worsening wooden breast severity, the proportional total myofiber area markedly decreased, reaching 61% in the superficial part and 82% in the deep part in severe wooden breast, while the proportional connective tissue + necrosis areas, respectively, increased to 27% (*p* < 0.01) and 7.7% (*p* > 0.05). A drastic change is also seen in the histological images (Figure 5), where connective tissue, necrotic and regenerating muscle fibers, and adipocytes gradually replace intact muscle. Concurrently, the remaining muscle fiber cross-sections change from angular to round as interstitial connective tissue separates them.

### 3.4. Protein Denaturation

**Figure 6:**
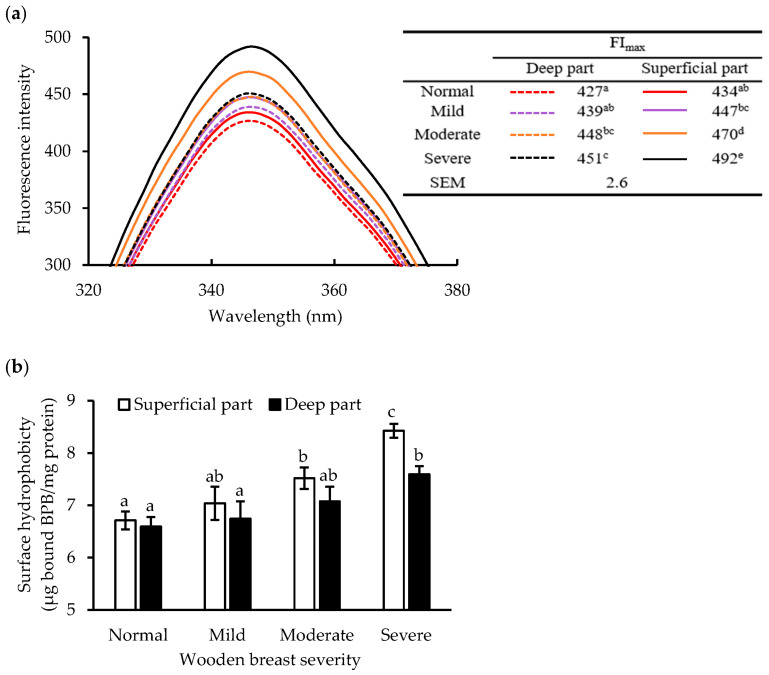
Effects of wooden breast severity on protein denaturation indicated by intrinsic fluorescence of sarcoplasmic proteins (**a**) and surface hydrophobicity of myofibrils (**b**) in the superficial and deep part of *pectoralis major* muscle. The tryptophan maximum fluorescence intensity (FI_max_) is summarized in the table embedded in Figure 6a. SEM: standard error of mean. Means with standard errors (*n* = 10) are shown in Figure 6b. BPB: bromophenol blue. a–e: within each indicator, mean values with the same letter do not differ (*p* > 0.05).

Muscle myopathies may lead to aberrant protein structures. In this experiment, intrinsic fluorescence and surface hydrophobicity were used to indicate the denaturation of sarcoplasmic and myofibrillar proteins, respectively. As shown in Table 1, intrinsic fluorescence and surface hydrophobicity were significantly affected by wooden breast severity (*p* < 0.01) and location within the muscle (*p* < 0.01). Clear changes in intrinsic fluorescence emission spectra on tryptophan residues from sarcoplasmic fractions with different wooden breast conditions are seen in Figure 6a, illustrating that more severe lesions resulted in higher emission spectra. In addition, the maximum fluorescence intensity (FI_max_) in both the superficial and deep parts from severely affected muscles increased, by 13.4% (*p* < 0.01) and 5.6% (*p* < 0.05), respectively. There was a significant interaction effect between wooden breast severity and location (*p* < 0.05, Table 1). With the development of the wooden breast condition, no significant shift in the maximum fluorescence emission wavelength (λ_max_) was observed (Figure 6a).

Figure 6b demonstrates how the surface hydrophobicity of myofibrils was affected by wooden breast severity. The surface hydrophobicity increased remarkably with the severity, especially in the superficial part, however, myofibrils from the severe group in both locations showed significantly higher values of surface hydrophobicity in comparison to the normal group. The markers of myofibrillar and sarcoplasmic protein denaturation showed similar trends, i.e., increasing denaturation with wooden breast severity and more pronounced denaturation in the superficial part, but with the exception that no interaction between wooden breast severity and location was observed regarding surface hydrophobicity (Table 1).

### 3.5. Pearson Correlations

Pearson correlation coefficients between the parameters of protein fractions, muscle areas, and protein denaturation are presented in Table 2. In the current study, significantly positive relationships were observed between total myofiber area and sarcoplasmic and myofibrillar proteins, with coefficients of 0.73 (*p* < 0.01) and 0.56 (*p* < 0.01), respectively. In addition, heat-insoluble collagen showed a negative correlation to total myofiber area (r = −0.55, *p* < 0.01) and a positive correlation to connective tissue + necrosis area (r = 0.52, *p* < 0.01), while no significant association was found with heat-soluble collagen. The protein denaturation indicators showed negative (*p* < 0.01) correlations to sarcoplasmic and salt-soluble myofibrillar protein, and the intrinsic fluorescence showed a high negative correlation to the myofiber area and a positive correlation to the connective tissue and necrosis area.

### 3.6. PCA Analysis

**Figure 7:**
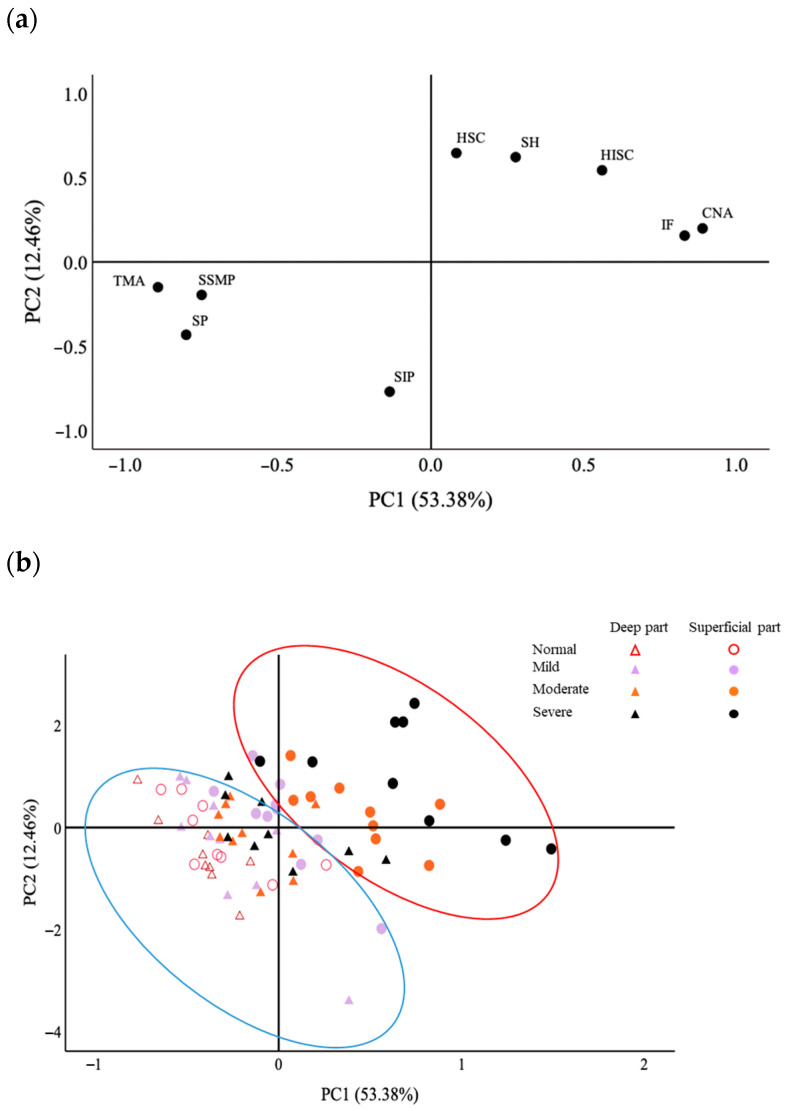
Principal component analysis of all factors of protein fractions, total myofiber area, and connective tissue + necrosis area as well as protein denaturation in wooden-breast-affected muscle. (**a**) The loading plot of all factors and (**b**) score distribution of samples based on two principal components. PC1: the first principal component, PC2: the second principal component. SP: sarcoplasmic protein, SSMP: salt-soluble myofibrillar protein, SIP: salt-insoluble protein, HSC: heat-soluble collagen, HISC: heat-insoluble collagen, TMA: total myofiber area, CNA: connective tissue + necrosis area, SH: surface hydrophobicity, IF: intrinsic fluorescence.

PCA was conducted by reducing the dimensionality of the input variables to reveal the distribution of the samples and better visualize the separation of different affected groups. In this study, the first principal component (PC1) explained 53% of the variance, and the second principal component (PC2) explained 12% (Figure 7). Thus, the two principal components accounted for about 65% of the total variance. The loading plot of factors and score distribution of samples based on the two principal components are shown in Figure 7.

Sarcoplasmic and myofibrillar protein content as well as the percentage of total myofiber area contributed highly negatively to PC1, showing loadings of −0.80, −0.75, and −0.90, respectively (Figure 7a). In contrast, heat-insoluble collagen, connective tissue + necrosis area, and intrinsic fluorescence contributed highly positively to PC1. Heat-soluble collagen and surface hydrophobicity presented a high positive loading on PC2, whereas salt-insoluble protein showed a high negative loading on PC2.

## 4. Discussion

### 4.1. Muscle Protein Changes

The results of the present study are consistent with the view that muscle fibers, especially in the superficial part, are gradually replaced by connective tissue with increasing development of wooden breast condition. Although decreased total protein content and increased collagen content in broiler chicken with wooden breast myopathy have been reported previously [10,25], this is to our knowledge the first study investigating the effect of wooden breast severity in combination with muscle location. In addition to providing information on the changes in muscle protein fractions, we here reveal the association behind their changes. In the current study, both wooden breast severity and location showed significant effects on the level of muscle protein fractions (Table 1), and the superficial part was demonstrated to have less sarcoplasmic proteins, salt-soluble myofibrillar proteins, and salt-insoluble proteins than the deep part at severe wooden breast (Figure 2). Our results are consistent with previous studies: Bowker et al. [26] extracted 92.2 mg/g sarcoplasmic protein from normal and 74.4 mg/g from severe wooden breast muscle but found no significant change in myofibrillar protein. Soglia et al. [10] found by applying SDS-PAGE that the relative abundance of bands of the sarcoplasmic and myofibrillar proteins were significantly lower in wooden breast. Decreased muscle protein fractions in wooden breast muscle may contribute to the changes in total protein content (calculated from the sum of sarcoplasmic and myofibrillar proteins) that declined from 23.1% to 16.7% in the superficial part and from 24.3% to 21.0% in the deep part. This is also consistent with Wold et al. [27], who reported that the total protein content in the upper 1 cm of breast muscle (superficial part) reduced from 23.5% in the normal muscle to 18.4% in the severe wooden-breast-affected muscle. Sihvo et al. [3] found a reduction in muscle fiber number due to the chronic myodegeneration and replacement of the necrotic muscle fibers with connective tissue in wooden breast muscles, and we concluded that this will consequently result in a decrease in sarcoplasmic and myofibrillar protein contents.

Collagen fractions, especially heat-insoluble collagen, in contrast to myofibrillar protein, increased with wooden breast severity (Figure 3b). In agreement with previous studies [13,28], the collagen to total protein ratio increased in wooden-breast-affected muscles, and there was a significantly higher content of heat-insoluble collagen, while no remarkable change in soluble collagen was observed. The increasing collagen content in the affected muscles may be attributed to altered muscle architecture, as degenerated fibers are replaced by connective tissue (Figure 5). Heat-insoluble collagen content increased significantly, which may be associated with the greater degree of crosslinks in collagen fibrils. This has been shown to be characterized by mature, thermal stable, non-reducible crosslinks that are more likely formed in fibrotic myopathies, evidenced by a relatively higher level of collagen crosslinking, indicated by hydroxylysylpyridinoline crosslinks and its regulator’s expression in wooden breast abnormality [8,29]. Accumulation of such collagenous connective tissue can contribute to the formation of larger particle sizes upon muscle homogenization, thereby protecting myofibrils against fragmentation [4], and additionally resulting in lower protein salt-solubility [30]. This could also explain the negative correlations between collagen fractions and the other protein fractions (Table 2). Although “salt-soluble” is often used to describe myofibrillar protein, not all myofibrillar proteins are extractable or salt-soluble [11], the salt-insoluble protein fraction thus contains some myofibrillar proteins in addition to connective tissue protein. The decreased salt-insoluble protein content with wooden breast severity (Figure 2c) suggests that the myofibrillar protein degradation rate in wooden-breast-affected muscle is higher than the connective tissue accumulation rate.

Overall, the loss of myofibrillar protein and gradual increase in collagen content caused by altered muscle fiber architecture was also consistent with our histological observations, and the changes in superficial muscle were more pronounced than in deep muscle (Figure 5), indicating that the surface layer is more prone to lesions in wooden breast [4]. Furthermore, in our study, fibrosis was subjectively more prominent in severe wooden breast as previously reported [3]. Changes in protein composition and integrity may impair the interaction of proteins with other biomolecules [2]. Therefore, the modifications of protein composition, such as changes in the amount and solubility of sarcoplasmic and myofibrillar proteins shown in this study, may result in lowered muscle functionality even in connection with mild myodegeneration. This includes decreased water-holding capacity, which is likely associated with reduced sarcoplasmic and myofibrillar protein content in abnormal meat [12]. In addition, the accumulation of heat-stable collagen might contribute to the toughness of wooden breast meat [8].

### 4.2. Protein Denaturation

We evaluated wooden-breast-induced conformational changes in sarcoplasmic and myofibrillar proteins using intrinsic fluorescence spectra of tryptophan residues and surface hydrophobicity, respectively. The intensity of tryptophan fluorescence is highly sensitive to its surrounding micro-environment, and thus changes in FI_max_ and λ_max_ can reflect the conversion of protein folding/unfolding [31]. There was no significant shift in λ_max_, indicating that wooden breast did not exert significant alterations on the micro-environment surrounding tryptophan residues in the sarcoplasmic protein. However, the FI_max_ significantly increased with wooden breast severity (Figure 6a), suggesting that tryptophan residues in sarcoplasmic protein of the affected muscle were exposed to a more nonpolar environment [32]. Here, we speculate that interactions between proteins and other biomolecules associated with muscle fiber degeneration and necrosis caused a less polar environment for tryptophan residues, due to an increased aggregation of denatured sarcoplasmic proteins, which consequently resulted in the increase in FI_max_. In addition, a considerably higher surface hydrophobicity of myofibrils was observed in severe wooden breast, showing that the buried hydrophobic amino acids were exposed to the filament surface, which evidenced that also the myofibrillar protein changed conformation with the development of wooden breast. In general, protein denaturation also reduces protein solubility [33]. Accordingly, we found strong negative correlations between protein denaturation indicators and sarcoplasmic and myofibrillar protein contents. Therefore, reduced solubility of sarcoplasmic and myofibrillar protein could also be related to their denaturation.

### 4.3. Protein Alterations in Association to Wooden Breast Development

Combining information on total myofiber and connective tissue + necrosis areas with protein measurements may help to understand in more detail why the content of sarcoplasmic and myofibrillar proteins as well as their solubilities are reduced in wooden breast muscle. We showed that the proportion of total myofiber area deceased while connective tissue + necrosis area increased markedly with increasing wooden breast severity, in both superficial and deep breast muscle (Figure 5), and the reduced total myofiber area in our study most likely also indicates a decrease in fiber number [34]. In agreement, a wooden breast-myopathy-induced reduction in muscle fiber number has been reported by Sihvo et al. [3] and Dalle Zotte et al. [35]. Compromised microcirculation leading to a reduction in oxygen supply and impaired waste removal in muscle fibers has been suggested to induce myodegeneration, initiate cell necrosis, and further promote collagen synthesis, and eventually result in wooden breast myopathy [2]. With the worsening wooden breast, progressive degeneration of muscle fibers and the accumulation of connective tissue lead to the observed decline in total myofiber and the reverse increase in connective tissue and degenerated or necrotic muscle fiber areas (Figure 5). The significantly positive correlations between total myofiber proportion and sarcoplasmic protein content (r = 0.73; *p* < 0.01) and salt-insoluble myofibrillar protein content (r = 0.56; *p* < 0.01) suggest that changes in these protein fractions in wooden breast muscle are associated with a reduction in total myofiber area. The disruption of sarcolemma caused by fiber degeneration can lead to the leakage of sarcoplasm and alteration of the protein profile [36]. Mudalal et al. [12] and Abasht et al. [37] found reduced levels of glycolytic enzymes among sarcoplasmic proteins in white striping and wooden breast abnormal chicken breast, respectively. In addition, possible sarcomere damage and disorganization in wooden breast [38], such as the observed lack of nebulin in the superficial layer of wooden breast muscle [39], may subsequently result in increased vulnerability to proteases followed by degradation of myofibrillar proteins [40]. Therefore, we believe that in our study the overall loss in protein content and solubility emanate from an altered protein profile and increased protein degradation and denaturation, which for their part stem mainly from myodegeneration. Furthermore, the negative associations between total myofiber area and the myofibrillar (r = −0.25, *p* < 0.05) and sarcoplasmic (r = −0.67, *p* < 0.01) protein denaturation indicators show that ongoing degradation and denaturation of structural proteins may also contribute to muscle fiber damage and consequently aggravate muscle fiber reduction.

In the principal component analysis, the loading contributions of the individual indictors (Figure 7a) appear to show that PC1 mainly involves protein and myofiber proportions and PC2 collagen content and protein denaturation. Total myofiber area, sarcoplasmic protein, and salt-soluble myofibrillar protein variables group together, suggesting both close correlation and similar wooden breast severity effects for these three variables. In Figure 7, the two principal components arranged all the indicators into two distinct groups. Group 1 (blue oval) is related to the normal muscle condition, showing a higher level of sarcoplasmic and salt-soluble myofibrillar proteins, and total myofiber area. Group 2 (red oval) is related to abnormal muscle, positively correlating with collagen content, connective tissue + necrosis area, and protein denaturation. With the development of wooden breast severity, the score distributions of samples gradually moves from group 1 to group 2 (Figure 7b). This implies that the wooden breast development is positively linked to the amount of collagen, protein denaturation, and fiber necrosis. Moreover, the sample scores of the superficial part show more pronounced movement toward group 2 than the deep part, indicating that the superficial part of the breast muscle is more prone to wooden breast myodegeneration than the deep part, confirming the findings of Soglia et al. [4]. In agreement, Maxwell et al. [41] analyzed non-marinated and marinated breast fillet portions and found that the effects of wooden breast on texture attributes (such as hardness, cohesiveness, and fibrousness) were more apparent in the ventral portions (superficial part) of the fillets than in the dorsal portions (deep part).

## 5. Conclusions

In conclusion, the superficial part of the breast muscle is more prone to wooden breast myodegeneration than the deep part. Increasing severity of the chronic myodegeneration leads to advancing muscle cell necrosis and a reduced area of muscle fibers that are related to an extensive decrease in sarcoplasmic, salt-soluble myofibrillar, and salt-insoluble proteins, and an overall decline in muscle protein. Muscle cell loss and reduced muscle fiber area lead to interstitial connective tissue accumulation that is positively linked to increased collagen content and sarcoplasmic and myofibrillar protein denaturation. Based on this, we propose that the lower overall content of protein in wooden-breast-affected muscle is mainly due to a reduction in the total myofiber volume. In addition, there is a high degree of protein denaturation induced by muscle fiber degeneration accompanied by connective tissue accumulation and fibrosis.

## Figures and Tables

**Table 1:** Main effects of wooden breast severity, location, and their interaction effects on the protein contents of muscle fractions, muscle subsection areas, and protein denaturation indicators.

Effects	WB Severity	Location	WB Severity *
			Location
Protein fractions			
SP	**	**	**
SSMP	**	**	ns
SIP	*	*	ns
HSC	ns	**	ns
HISC	**	**	*
Muscle subsection areas			
TMA	**	**	**
CNA	**	**	**
Protein denaturation indicators			
SH	**	*	ns
IF	**	**	*

ns: not significant. SP: sarcoplasmic protein, SSMP: salt-soluble myofibrillar protein, SIP: salt-insoluble protein, HSC: heat-soluble collagen, HISC: heat-insoluble collagen, TMA: total myofiber area, CNA: connective tissue + necrosis area, SH: surface hydrophobicity, IF: intrinsic fluorescence. * *p* < 0.05; ** *p* < 0.01.

**Table 2:** Pearson correlation between the protein contents of muscle fractions, muscle subsection areas, and protein denaturation indicators in wooden-breast-affected muscle.

	SP	SSMP	SIP	HSC	HISC	TMA	INA	SH	IF
SP	1								
SSMP	0.71 **	1							
SIP	0.39 **	0.10	1						
HSC	−0.26 *	−0.27 *	−0.23 *	1					
HISC	−0.64 **	−0.58 **	−0.33 **	0.39 **	1				
TMA	0.73 **	0.56 **	0.27 *	−0.27 *	−0.55 **	1			
CNA	−0.76 **	−0.55 **	−0.35 **	0.29 *	0.52 **	−0.95 **	1		
SH	−0.57 **	−0.34 **	−0.27 *	0.19	0.41 **	−0.25 *	0.28 *	1	
IF	−0.71 **	−0.62 **	−0.18	0.16	0.45 **	−0.67 **	0.71 **	0.49 **	1

SP: sarcoplasmic protein, SSMP: salt-soluble myofibrillar protein, SIP: salt-insoluble protein, HSC: heat-soluble collagen, HISC: heat-insoluble collagen, TMA: total myofiber area, CNA: connective tissue + necrosis area, SH: surface hydrophobicity, IF: intrinsic fluorescence. * *p* < 0.05; ** *p* < 0.01.

## Data Availability

Data is contained within the article.
